# Supplementary material for: Effects of Adding Blueberry Residue Powder and Extrusion Processing on Nutritional Components, Antioxidant Activity and Volatile Organic Compounds of Indica Rice Flour
Source: Biology (Basel). 2022 Dec 14;11(12):1817. doi: 10.3390/biology11121817 (PMC9775675; doi:10.3390/biology11121817)
Supplement: Supplementary file 1 [file biology-11-01817-s001.zip › biology-1990989-supplementary.pdf]

Supplementary Materials:

Table S1. GC-IMS integration parameters of volatile compounds detected in indica rice expanded powder and blueberry residue expanded powder by GC-IMS.

Count	Compound	CAS#	Formula	MW	Classification	RI	Rt [sec]	Dt [RIPrel]	Signal Intensity		p-Value
									IREP	BREP	
1	gamma-Butyrolactone	C96480	C4H6O2	86.1	Esters	1638.3	1715.984	1.11009	815.02 ±245.57[b]	3657.03 ±832.99[a]	0.005
2	2-Methylpropanoic acid	C79312	C4H8O2	88.1	Acids	1632.3	1693.762	1.15455	229.18 ±30.23[b]	786.41 ±154.56[a]	0.004
3	Ethyl 3-hydroxybutanoate	C5405414	C6H12O3	132.2	Esters	1597.2	1569.319	1.16391	541.27 ±65.91[a]	370.05 ±55.68[b]	0.026
4	(E)-2-Nonenal	C18829566	C9H16O	140.2	Aldehydes	1572.1	1486.357	1.41897	349.22 ±8.05[b]	536.13 ±64.06[a]	0.07
5	Benzaldehyde	C100527	C7H6O	106.1	Aromatic Aldehydes	1546.6	1406.358	1.15923	626.53 ±2.14[a]	526.99 ±47.81[b]	0.023
6	Decanal	C112312	C10H20O	156.3	Aldehydes	1527.3	1348.58	1.54533	1277.63 ±85.53[a]	734.51 ±35.38[b]	0.001
7	Acetic acid-M	C64197	C2H4O2	60.1	Acids	1502.9	1278.951	1.06095	4335.09 ±194.11[b]	17111.55 ±736.58[a]	<0.001
8	Acetic acid-D	C64197	C2H4O2	60.1	Acids	1503.4	1280.433	1.15923	482.40 ±29.39[b]	11668.89 ±1570.20[a]	<0.001
9	1-Octen-3-ol	C3391864	C8H16O	128.2	Alcohols	1482.7	1224.137	1.17093	187.02 ±23.88[b]	227.02 ±25.45[a]	0.118
10	(E)-2-Octenal	C2548870	C8H14O	126.2	Aldehydes	1437.6	1110.064	1.33941	219.93 ±23.11[b]	364.41 ±20.18[a]	0.001
11	Nonanal-M	C124196	C9H18O	142.2	Aldehydes	1403.0	1029.714	1.47979	4479.82 ±133.81[b]	3567.87 ±17.78[a]	<0.001
12	Nonanal-D	C124196	C9H18O	142.2	Aldehydes	1403.0	1029.714	1.94733	813.76 ±64.22[a]	516.47 ±21.17[b]	0.002
13	1-Hexanol	C111273	C6H14O	102.2	Alcohols	1368.6	955.592	1.32951	147.98 ±3.73[b]	181.69 ±6.88[a]	0.002
14	6-Methyl-5-hepten-2-one	C110930	C8H14O	126.2	Ketones	1347.3	912.466	1.18294	243.09 ±24.29[b]	472.25 ±24.54[a]	<0.001
15	(E)-Ethyl-2-hexenoate	C27829727	C8H14O2	142.2	Esters	1348.0	913.814	1.3258	82.61 ±1.26[b]	101.55 ±7.69[a]	0.014
16	1-Hydroxy-2-propanone	C116096	C3H6O2	74.1	Ketones	1314.1	849.125	1.04936	567.19 ±17.59[b]	937.88 ±142.83[a]	0.011
17	Acetoin-M	C513860	C4H8O2	88.1	Ketones	1298.5	820.823	1.05678	299.08 ±12.21[b]	1736.98 ±198.58[a]	<0.001
18	Acetoin-D	C513860	C4H8O2	88.1	Ketones	1300.0	823.519	1.33508	36.27 ±1.46[b]	351.47 ±74.50[a]	0.002
19	(E)-2-Heptenal-M	C18829555	C7H12O	112.2	Aldehydes	1331.4	881.469	1.25901	899.69 ±22.71[b]	1180.24 ±105.37[a]	0.011
20	(E)-2-Heptenal-D	C18829555	C7H12O	112.2	Aldehydes	1332.1	882.817	1.6746	95.53 ±10.96[b]	135.88 ±18.19[a]	0.030

21	2-Heptanol	C543497	C7H16O	116.2	Alcohols	1330.6	880.121	1.38332	564.26 ±24.69[a]	448.41 ±47.49[b]	0.020
22	Ethyl heptanoate	C106309	C9H18O2	158.2	Esters	1345.2	908.423	1.40929	118.57 ±7.11[b]	146.38 ±8.94[a]	0.014
23	2,3-Dimethylpyrazine	C5910894	C6H8N2	108.1	Heterocyclics	1336.3	890.903	1.09574	295.42 ±56.84[a]	198.56 ±23.52[b]	0.053
24	2,5-Dimethylpyrazine	C123320	C6H8N2	108.1	Heterocyclics	1313.4	847.777	1.10873	365.91 ±13.95[a]	361.47 ±23.83[a]	0.794
25	1-Octen-3-one	C4312996	C8H14O	126.2	Ketones	1311.2	843.734	1.27756	203.22 ±15.19[b]	321.28 ±20.74[a]	0.001
26	Hexyl acetate	C142927	C8H16O2	144.2	Esters	1313.4	847.777	1.39074	206.34 ±14.91[a]	193.11 ±11.86[a]	0.296
27	Cyclohexanone	C108941	C6H10O	98.1	Ketones	1298.5	820.823	1.16439	273.74 ±8.75[b]	1501.58 ±180.87[a]	<0.001
28	Octanal	C124130	C8H16O	128.2	Aldehydes	1299.3	822.171	1.40372	557.55 ±48.15[b]	660.03 ±38.56[a]	0.045
29	Pentanol	C71410	C5H12O	88.1	Alcohols	1264.5	764.68	1.25664	205.20 ±18.69[b]	485.25 ±72.00[a]	0.003
30	Ethyl hexanoate	C123660	C8H16O2	144.2	Esters	1246.1	736.154	1.34209	228.44 ±16.04[b]	705.83 ±68.34[a]	<0.001
31	2-Pentylfuran	C3777693	C9H14O	138.2	Aromatic heterocyclics	1243.1	731.553	1.25517	86.60 ±4.19[b]	416.77 ±11.47[a]	<0.001
32	(E)-2-Hexenal	C6728263	C6H10O	98.1	Aldehydes	1233.3	716.83	1.18445	172.83 ±16.23[b]	466.37 ±30.22[a]	<0.001
33	3-Octanone	C106683	C8H16O	128.2	Ketones	1232.6	715.91	1.30084	186.14 ±4.79[b]	449.01 ±31.33[a]	<0.001
34	2-Methyl-1-butanol	C137326	C5H12O	88.1	Alcohols	1228.9	710.389	1.47174	144.76 ±8.51[b]	270.41 ±31.44[a]	0.003
35	3-Methyl-1-butanol	C123513	C5H12O	88.1	Alcohols	1222.0	700.267	1.2478	212.58 ±19.59[b]	869.75 ±55.53[a]	<0.001
36	Heptanal-M	C111717	C7H14O	114.2	Aldehydes	1197.2	665.3	1.32736	732.24 ±45.14[b]	1042.59 ±37.59[a]	0.001
37	Heptanal-D	C111717	C7H14O	114.2	Aldehydes	1197.2	665.3	1.69862	84.78 ±11.37[b]	245.69 ±16.24[a]	<0.001
38	Methyl hexanoate-M	C106707	C7H14O2	130.2	Esters	1189.4	652.417	1.26843	768.10 ±71.60[b]	1725.25 ±70.01[a]	<0.001
39	Methyl hexanoate-D	C106707	C7H14O2	130.2	Esters	1188.9	651.497	1.68831	180.63 ±13.75[b]	968.91 ±66.39[a]	<0.001
40	1-Penten-3-ol	C616251	C5H10O	86.1	Alcohols	1176.8	625.732	0.94431	597.82 ±42.42[b]	1125.03 ±51.23[a]	<0.001
41	Myrcene	C123353	C10H16	136.2	Terpenoids	1177.2	626.652	1.21392	91.39 ±5.00[b]	291.68 ±3.90[a]	<0.001
42	Pentyl acetate	C628637	C7H14O2	130.2	Esters	1179.9	632.173	1.31557	141.40 ±31.42[a]	128.38 ±8.24[a]	0.526
43	1-Butanol	C71363	C4H10O	74.1	Alcohols	1161.8	595.365	1.18298	973.34 ±46.57[a]	806.89 ±12.82[b]	0.004
44	Butyl propionate-M	C590012	C7H14O2	130.2	Esters	1156.6	585.243	1.28463	147.33 ±14.93[b]	514.47 ±26.69[a]	<0.001
45	Butyl propionate-D	C590012	C7H14O2	130.2	Esters	1156.6	585.243	1.72219	31.82 ±1.49[b]	171.22 ±17.93[a]	<0.001

46	Cyclopentanone-M	C120923	C5H8O	84.1	Ketones	1147.5	567.76	1.10932	3131.76 ±175.31[a]	1757.89 ±119.87[b]	<0.001
47	Cyclopentanone-D	C120923	C5H8O	84.1	Ketones	1146.5	565.919	1.32883	3236.73 ±327.61[a]	1401.86 ±92.74[b]	0.001
48	Isoamyl acetate-M	C123922	C7H14O2	130.2	Esters	1138.1	550.276	1.30231	1889.86 ±57.92[b]	2430.49 ±40.79[a]	<0.001
49	Isoamyl acetate-D	C123922	C7H14O2	130.2	Esters	1138.1	550.276	1.74871	2074.99 ±111.82[b]	6358.44 ±138.56[a]	<0.001
50	beta-Pinene	C127913	C10H16	136.2	Terpenoids	1150.6	573.703	1.21785	52.96 ±1.12[b]	90.79 ±2.92[a]	<0.001
51	2-Methyl-1-propanol	C78831	C4H10O	74.1	Alcohols	1108.8	499.278	1.16977	365.54 ±25.83[b]	435.86 ±22.09[a]	0.023
52	Hexanal-M	C66251	C6H12O	100.2	Aldehydes	1101.0	486.572	1.25991	4221.77 ±29.48[b]	4365.67 ±25.18[a]	0.003
53	Hexanal-D	C66251	C6H12O	100.2	Aldehydes	1101.4	487.177	1.5649	6751.54 ±30.15[b]	9312.36 ±82.80[a]	<0.001
54	Butyl acetate-M	C123864	C6H12O2	116.2	Esters	1087.8	467.209	1.23738	685.06 ±22.05[b]	1381.06 ±43.79[a]	<0.001
55	Butyl acetate-D	C123864	C6H12O2	116.2	Esters	1088.2	467.814	1.61748	213.64 ±10.63[b]	1174.36 ±67.93[a]	<0.001
56	Dimethyl disulfide	C624920	C2H6S2	94.2	Ethers	1084.7	462.973	1.1247	125.79 ±8.55[b]	421.02 ±11.01[a]	<0.001
57	Ethyl butanoate-M	C105544	C6H12O2	116.2	Esters	1052.8	421.223	1.20583	2935.30 ±15.50[a]	2682.41 ±15.79[b]	<0.001
58	Ethyl butanoate-D	C105544	C6H12O2	116.2	Esters	1052.8	421.223	1.5634	11127.09 ±91.83[b]	16170.03 ±41.17[a]	<0.001
59	1-Penten-3-one-M	C1629589	C5H8O	84.1	Ketones	1042.6	408.697	1.07996	172.88 ±2.32[b]	357.96 ±4.67[a]	<0.001
60	1-Penten-3-one-D	C1629589	C5H8O	84.1	Ketones	1041.6	407.419	1.31199	30.43 ±1.70[b]	130.51 ±4.15[a]	<0.001
61	Methyl 2-methylbutanoate	C868575	C6H12O2	116.2	Esters	1014.0	375.458	1.19114	408.66 ±12.80[b]	610.94 ±14.67[a]	<0.001
62	Isobutyl acetate	C110190	C6H12O2	116.2	Esters	1022.7	385.259	1.23706	106.31 ±7.23[b]	283.63 ±9.14[a]	<0.001
63	Methyl 3-methylbutanoate	C556241	C6H12O2	116.2	Esters	1041.6	407.419	1.19114	160.63 ±11.09[b]	319.44 ±8.38[a]	<0.001
64	4-Methyl-2-pentanone	C108101	C6H12O	100.2	Ketones	1031.2	395.061	1.17906	237.33 ±9.59[a]	237.56 ±6.39[a]	0.974
65	Ethyl 2-methylbutanoate	C7452791	C7H14O2	130.2	Esters	1039.1	404.436	1.22377	145.85 ±13.90[a]	135.80 ±10.61[b]	0.376
66	Pentanal	C110623	C5H10O	86.1	Aldehydes	1003.1	363.526	1.42559	526.08 ±17.43[b]	1051.45 ±13.65[a]	<0.001
67	2-Pentanone	C107879	C5H10O	86.1	Ketones	1000.4	360.543	1.38933	296.18 ±14.70[b]	607.26 ±9.63[a]	<0.001
68	Ethanol	C64175	C2H6O	46.1	Alcohols	944.1	316.651	1.12347	30046.90 ±383.69[a]	29222.34 ±286.45[b]	0.041
69	Propyl acetate	C109604	C5H10O2	102.1	Esters	969.7	335.401	1.47393	14.19 ±2.57[b]	57.64 ±2.66[a]	<0.001
70	3-Methylbutanal	C590863	C5H10O	86.1	Aldehydes	928.9	305.998	1.40263	706.16 ±14.89[b]	2820.40 ±19.52[a]	<0.001
71	tert-Butanol	C75650	C4H10O	74.1	Alcohols	933.9	309.407	1.32408	593.41 ±24.38[b]	1112.46 ±29.39[a]	<0.001

72	Diethyl acetal	C105577	C6H14O2	118.2	Aldehydes	909.3	292.787	1.03162	4112.79 ±57.93^{b}	5026.91 ±10.83^{a}	<0.001
73	2-Butanone	C78933	C4H8O	72.1	Ketones	917.7	298.327	1.24673	1707.38 ±79.06^{b}	2022.95 ±23.57^{a}	0.003
74	Ethyl Acetate	C141786	C4H8O2	88.1	Esters	896.2	284.265	1.33858	1243.40 ±46.74^{b}	2862.88 ±11.38^{a}	<0.001
75	Butanal	C123728	C4H8O	72.1	Aldehydes	890.2	280.429	1.11138	414.70 ±0.90^{b}	441.64 ±1.59^{a}	<0.001
76	Acrolein	C107028	C3H4O	56.1	Aldehydes	867.4	266.367	1.06062	272.02 ±10.68^{b}	510.62 ±19.04^{a}	<0.001
77	Methyl acetate	C79209	C3H6O2	74.1	Esters	852.9	257.844	1.19839	28.88 ±1.09^{b}	116.66 ±4.45^{a}	<0.001
78	Acetone	C67641	C3H6O	58.1	Ketones	839.5	250.174	1.1138	7628.98 ±188.01^{b}	8484.97 ±162.63^{a}	0.004
79	2-Methylpropanal	C78842	C4H8O	72.1	Aldehydes	830.4	245.06	1.27936	49.31 ±1.31^{b}	156.39 ±6.35^{a}	<0.001
80	Propanal-M	C123386	C3H6O	58.1	Aldehydes	820.2	239.52	1.05217	1215.67 ±15.00^{a}	1141.00 ±16.91^{b}	0.005
81	Propanal-D	C123386	C3H6O	58.1	Aldehydes	821.0	239.946	1.14643	1471.86 ±17.84^{b}	3476.80 ±4.90^{a}	<0.001
82	Dimethyl sulfide	C75183	C2H6S	62.1	Ethers	798.3	228.014	0.96032	257.11 ±1.75^{b}	651.39 ±34.72^{a}	<0.001
83	Acetaldehyde	C75070	C2H4O	44.1	Aldehydes	764.8	211.395	0.97724	1714.74 ±70.44^{b}	1840.92 ±40.72^{a}	0.055
84	Hexyl 2-methylbutyrate	C10032152	C11H22O2	186.3	Esters	1465.7	1179.86	1.54448	186.86 ±8.67^{a}	134.86 ±11.39	0.003
85	Ethyl formate	C109944	C3H6O2	74.1	Esters	828.9	244.267	1.21492	53.33 ±1.84^{b}	164.19 ±5.88^{a}	<0.001
86	(E)-2-Pentenal	C1576870	C5H8O	84.1	Aldehydes	1150.2	572.808	1.35797	52.88 ±6.48^{b}	128.76 ±8.37^{a}	<0.001

D: dimer, M: monomer. Means with different letters within a row differ significantly ($p < 0.05$).**RI** Represents the retention index in the capillary GC column. **Rt** Represents the retention time in the capillary GCcolumn. **Dt** Represents the drift time in the drift tube.
